# Dynamics of neocortical networks: connectivity beyond the canonical microcircuit

**DOI:** 10.1007/s00424-023-02830-y

**Published:** 2023-06-20

**Authors:** Heiko J. Luhmann

**Affiliations:** Institute of Physiology, University Medical Center of the Johannes Gutenberg University, Duesbergweg 6, D-55128 Mainz, Germany

**Keywords:** Cerebral cortex, Connections, Cell types, Synaptic function, Review

## Abstract

The neocortical network consists of two types of excitatory neurons and a variety of GABAergic inhibitory interneurons, which are organized in distinct microcircuits providing feedforward, feedback, lateral inhibition, and disinhibition. This network is activated by layer- and cell-type specific inputs from first and higher order thalamic nuclei, other subcortical regions, and by cortico-cortical projections. Parallel and serial information processing occurs simultaneously in different intracortical subnetworks and is influenced by neuromodulatory inputs arising from the basal forebrain (cholinergic), raphe nuclei (serotonergic), locus coeruleus (noradrenergic), and ventral tegmentum (dopaminergic). Neocortical neurons differ in their intrinsic firing pattern, in their local and global synaptic connectivity, and in the dynamics of their synaptic interactions. During repetitive stimulation, synaptic connections between distinct neuronal cell types show short-term facilitation or depression, thereby activating or inactivating intracortical microcircuits. Specific networks are capable to generate local and global activity patterns (e.g., synchronized oscillations), which contribute to higher cognitive function and behavior. This review article aims to give a brief overview on our current understanding of the structure and function of the neocortical network.

## Introduction

Understanding the circuit dynamics of a neuronal network requires a detailed knowledge of the structural and functional properties of its neuronal cell types, the underlying connectivity, and the state-dependent dynamics of the synaptic connections in the network. In invertebrates and in small neuronal networks consisting of a few hundreds of neurons, as in *Caenorhabditis elegans*, this seems to be a feasible task, although currently far from being solved. Even the most detailed anatomical connectome does not provide any information on the biophysical properties of the synaptic interactions, e.g., excitatory, inhibitory, strength, silent, transmitter release properties, receptor desensitization, and transmitter uptake mechanism. Therefore, it seems to be impossible to reconstruct in detail the cellular structure and function of a larger neuronal network, such as the circuits in the cerebral cortex of mammals. However, in 1989, Rodney Douglas, Kevan Martin, and David Whitteridge published a study entitled “A Canonical Microcircuit for Neocortex” [3], which became a landmark paper in the field. This study is based on the morphological and electrophysiological characterization of single neurons recorded intracellularly in primary visual cortex of anesthetized cats and the authors propose a basic wiring diagram of the thalamocortical inputs and intracortical connections between two populations of excitatory neurons (located in layer [L] 2-4 and L5-6) and one population of inhibitory neurons (for complete list of abbreviations, see Table 1). Over the last 30 years, simultaneous patch-clamp recordings from 2-8 synaptically connected neurons, immunohistochemical and genetic characterizations, viral tracing techniques, opto- and chemogenetic experimental manipulations, detailed (3D) reconstructions of the dendritic arborization, and axonal projections (although long-range axons are difficult to trace) provided a deeper understanding of the neocortical network [9, 16, 10].

**Table 1:** Table of abbreviations

Abbreviation	Full name
ACh	Acetylcholine
BpC	Bipolar cell
BsC	Basket cell
ChC	Chandelier cell
CRC	Cajal-Retzius cell
DBC	Double-bouquet cell
FO	First order
GABA	Gamma-aminobutyric acid
HO	Higher order
L	Layer
MC	Martinotti cell
NgfC	Neurogliaform cell
P	Parvalbumin
PC	Pyramidal cell
R	Reelin
S, SOM	Somatostatin
SC	Spinal cord
spSC	Spiny stellate cell
V	Vasoactive intestinal polypeptide
Y	Neuropeptide Y

## Neocortical cell types and connections

**Fig. 1:**
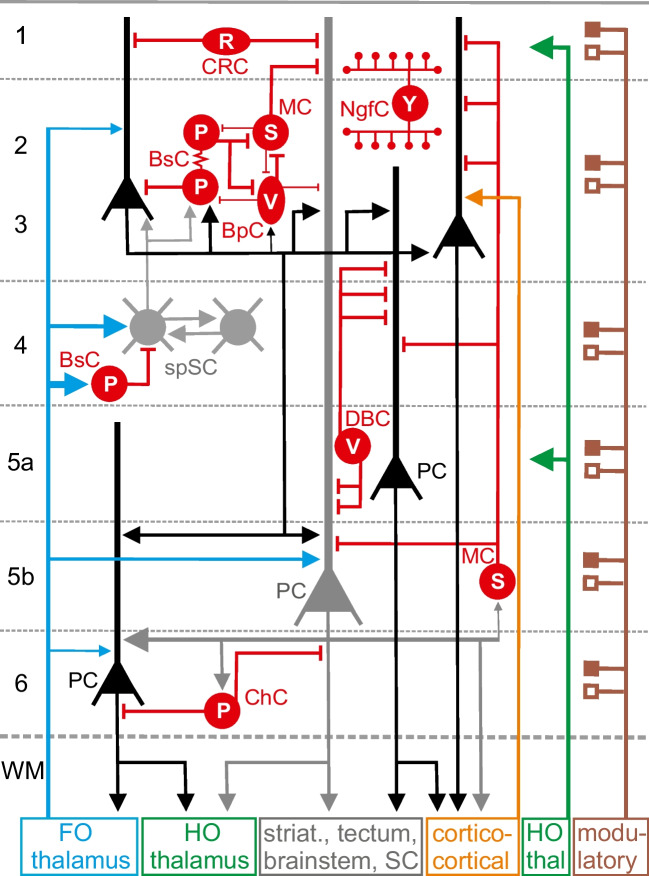
Schematic illustration of the neocortical microcircuitry with main afferent and efferent connections. Excitatory synaptic connections are represented by  , inhibitory by  ; electrical synapses by  , and volume transmission by  . Depolarizing and hyperpolarizing neuromodulatory inputs (depending on expression of postsynaptic receptors) are illustrated by  and  , respectively. Line thickness and arrow size indicate strength of synaptic connection, but for many connections, data are not available yet. Excitatory glutamatergic pyramidal cells (PCs) and spiny stellate cells (spSCs) are shown in black and gray. The population of inhibitory GABAergic neurons is shown in red and consists of Cajal-Retzius cells (CRC) expressing reelin (R), parvalbumin (P) expressing basket cells (BsC) and chandelier cells (ChC), vasoactive intestinal polypeptide (V) expressing bipolar cells (BpC) and double-bouquet cells (DBC), Martinotti cells (MC) expressing somatostatin (S), and neurogliaform cells (NgfCs) expressing neuropeptide Y (Y). Inhibitory interneurons may express also other markers (see text). Note that GABAergic neurons not only form axo-somatic connections, but also terminate on dendritic compartments or on the axon initial segment of pyramidal neurons. NgfCs often act through volume transmission and do not form synaptic connections with postsynaptic targets. Layer-specific glutamatergic inputs from first-order (FO) and higher order (HO) thalamus are shown in blue and green, respectively; ipsi- and contralateral cortico-cortical connections in orange color. Activating and inhibiting modulatory inputs from various subcortical sources to all cortical layers are illustrated by brown color. Pyramidal neurons project in a layer-dependent manner to the striatum, tectum, brainstem, spinal cord (SC), and to ipsi- and contralateral cortex. For clarity, not all connections are shown. Examples of local micro-networks consisting of excitatory and various types of inhibitory neurons are illustrated in the upper left part

Figure 1 gives a very schematic illustration of our current view on the intracortical connectivity and the main afferent and efferent connections of the cerebral cortex. This circuit diagram is largely based on in vitro and in vivo electrophysiological recordings in rodent and cat cerebral cortex. Over the last years, we learned that the human neocortex shows an increased diversity in neuronal cell types when compared to the rodent cortex [1]. Further electrophysiological and cellular imaging studies are required to understand the neuronal network of the human neocortex.

The population of glutamatergic excitatory neurons consists of pyramidal cells (PCs) and spiny stellate cells (spSCs) (for details on excitatory neuronal connectivity, see Feldmeyer [4]). The cell body of PCs can be located in every layer except L1 and the apical dendrite transverses several layers often forming an apical tuft in L1 and L2/3. SpSCs can be only found in L4 and the dendrite is mostly restricted to L4. Both cell types receive a glutamatergic input from first-order (FO) thalamic nuclei, such as ventral posterior nucleus and lateral or medial geniculate nucleus. Higher order (HO) thalamic nuclei, such as pulvinar and posterior nucleus, project to L1 and L5a. Only PCs form cortico-cortical connections and project to various subcortical targets, including FO thalamus innervated by feedback connections from L6 PCs (Fig. 1).

The population of GABAergic neurons is very diverse and far more complex than initially proposed by Douglas and colleagues in 1989. Figure 1 only shows the main subtypes of GABAergic cells as categorized by their morphology, function, genetics, and expression of molecular interneuron markers [2, 5, 22]. Although GABAergic cells comprise only 15–20% of all neocortical neurons, they play fundamental roles in cortical function [22] and dysfunction, e.g., psychiatric disorders [11]. Cajal-Retzius cells (CRCs) are only present in L1, express reelin, and target the apical dendrites of PCs. CRCs are most important in early cortical development, and their function in the adult cortex is not completely understood. Parvalbumin (PV) expressing basket cells (BsCs) are present in all layers, constitute the largest population of GABAergic interneurons, and innervate in a “basket”-like fashion the soma and perisomatic compartments of PCs. Some BsCs express cholecystokinin. BsCs are interconnected by chemical and electrical synapses and also synapse on other GABAergic cells forming feedback, disinhibitory, and lateral microcircuits. BsCs in L4 receive a strong thalamocortical input thereby providing feedforward inhibition to L4 spSCs and temporal precision of sensory inputs from FO thalamus. Furthermore, BsCs play important roles in network (gamma) oscillations.

Bipolar cells (BpCs) have a bipolar, vertically oriented dendrite crossing many layers; express vasointestinal peptide (Vip); and preferentially innervate somatostatin (Som) expressing interneurons, to a lesser extent PCs and BsCs. Double-bouquet cells (DBCs) represent a subtype of BpC also innervating dendrites of PCs. Martinotti cells (MCs) are found in L2-L6, express somatostatin, and form local and transcolumnar connections with PC dendrites. Other interneurons are only weakly innervated by MCs. Neurogliaform cells (NgfCs) express neuropeptide Y and have dense, spherical axonal projections, which often do not form classical synapses. NgfCs most likely act via volume transmission of GABA, thereby activating extrasynaptic receptors. Parvalbumin-expressing Chandelier cells (ChCs), also known as axo-axonic interneurons, form candlestick-like axonal projections, which innervate the axon initial segment of PCs. The intracortical microcircuitry in the upper left part of Figure 1 illustrates the complex network of feedforward and feedback, somatic and dendritic inhibition, and disinhibition. Similar inhibitory micronetworks can be found in lower layers.

In sensory cortical areas, this network may be organized in structural and functional cortical columns [16]. However, the concept of the cortical column (with many different types of columns) is not well defined and currently critically discussed [13]. Further, Figure 1 shows a schematic illustration of the various cell types and the basic connectivity pattern in the cerebral cortex. However, area- and species-specific modifications of this pattern have been reported [7]. For example, L4 in adult mouse visual and somatosensory cortex differs in excitatory and inhibitory cell types and intralaminar connectivity [17]. All cortical layers receive neuromodulatory inputs from the basal forebrain (cholinergic), raphe nuclei (serotonergic), locus coeruleus (noradrenergic), and ventral tegmentum (dopaminergic), which profoundly influence the function of excitatory and inhibitory neurons and networks. After summarizing the afferent, efferent, and intracortical anatomical connections, the next paragraph will describe the function of the neocortical neurons and the dynamics of synaptic interactions.

## Firing patterns and dynamics in synaptic functions

**Fig. 2:**
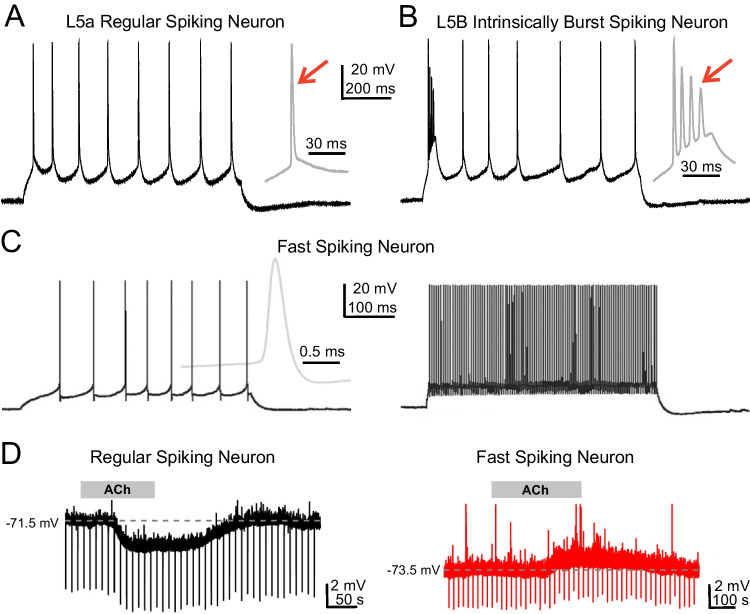
Neocortical neurons differ in their intrinsic discharge patterns and their responsiveness to acetylcholine. (**A**, **B**) Response of a L5a regular spiking (A) and a L5b intrinsically bursting pyramidal neuron (**B**) in rat somatosensory cortex to injection of a 150-pA current pulse. (**C**) Response of a fast spiking interneuron in monkey temporal cortex to 40-pA (left) and 200-pA (right) current injection. Resting membrane potential in A–C was −70 mV and initial discharge is shown at higher temporal resolution to the right. Note single spike discharge in (A) as compared to initial burst in (B) (red arrows) and brief action potential and high discharge frequency in (C). (**D**) Different response of a L4 regular spiking cell (left) and a L4 fast spiking neuron (right) to application of 30 μM acetylcholine (ACh). Hyperpolarizing current pulses were injected into the cell to monitor conductance changes. A and B reproduced from Schubert et al. [18]. C reproduced from Wang et al. [23] and D with permission from Qi et al. [14]

The action potential discharge pattern of a neuron depends on its passive (e.g., membrane potential, input resistance, membrane time constant) and active membrane properties (expression of voltage-dependent conductances) and can be broadly subdivided into regular firing, fast spiking, and burst firing (for additional firing patterns, see Markram et al. [12] and Tremblay et al. [22]). The intrinsic firing pattern determines how a neuron responds to suprathreshold synaptic activation and how this neuron will activate or inhibit its postsynaptic targets. Therefore, understanding the functional properties of a neuronal network requires the detailed characterization of the firing patterns of its neuronal elements. The intrinsic firing pattern of a single neuron can be studied by patch-clamp recording upon injection of short depolarizing current pulses of different intensity. Excitatory neurons show two basic types of discharge pattern. PCs and L4 spSCs generally show a regular spiking pattern consisting of single repetitive action potentials (~1 ms duration) (Fig. 2A). L5b PCs, characterized by their thick apical dendrite, and a small subpopulation of L4 spSCs respond to suprathreshold current injection with a burst consisting of an initial spike followed by a depolarizing afterpotential with action potentials of decreasing amplitude (Fig. 2B) [19, 18]. GABAergic neurons show a far more complex diversity in intrinsic firing patterns [10, 22]. PV-expressing BsCs and ChCs show brief spikes (~0.3 ms) and non-adapting discharges with frequencies up to 800 Hz (Fig. 2C). VIP-expressing BpCs and DBCs reveal regular, irregular (with small pauses), or fast spiking patterns with strong adaption. MCs expressing somatostatin show regular spiking, bursting, or low-threshold spiking (LTS) firing pattern. L1 CRCs reveal a regular spiking discharge. NgfCs expressing neuropeptide Y respond with a regular or irregular firing pattern. These findings demonstrate that neurons, especially inhibitory interneurons, are not simple analog-digital converters (as in electronic circuits) responding to suprathreshold synaptic activation with a homogeneous discharge pattern, but are rather complex units fulfilling in neocortical microcircuits very specific functions.

On the basis of detailed morphological and in vitro electrophysiological studies obtained in the somatosensory cortex of juvenile rats, Henry Markram and colleagues published a digital reconstruction of a neocortical microcircuit containing ~31,000 neurons and ~8 million connections with ~37 million synapses [12]. Although this model is based on electrophysiological recordings and 3D morphological reconstructions of neurons studied in 300-μm thin slice preparations, in which thalamic, cortico-cortical, and neuromodulatory inputs are largely not be preserved, the simulations in this model reproduced not only the observations of in vitro, but also of in vivo experiments in rodent cerebral cortex.

However, the impact of neuromodulatory inputs onto neocortical neurons and neuronal processing cannot be underestimated. Depending on the activation of receptor subtype, modulatory inputs have cell-type-specific de- or hyperpolarizing effects on their postsynaptic targets [14], thereby activating or inactivating neuronal microcircuits. Figure 2D shows an example how acetylcholine (ACh) can have a very different influence on neocortical neurons located in the same neocortical layer. Whereas application of ACh to a L4 regular spiking neuron causes a membrane hyperpolarization (left), the L4 fast spiking cell is depolarized by ACh (right). These data emphasize the role of the various neuromodulatory inputs on the functional state of single neurons, local microcircuits, and the control of cortical states like arousal and attention [8]. Further excitatory inputs arise from glutamatergic cortico-cortical inputs of the ipsi- and contralateral hemisphere, which become most powerful during oscillatory network activity. Distinct synaptic connections and local microcircuits, as shown in Fig. 1, can be rapidly switched on and off, thereby activating or inactivating local processing units.

**Fig. 3:**
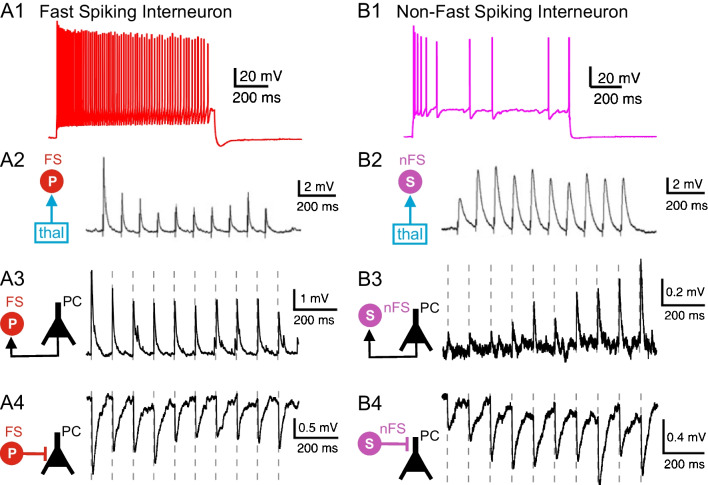
Firing pattern (upper row) and functional synaptic connectivity (rows 2–4) of a fast spiking parvalbumin expressing interneuron (**A**) and a non-fast spiking somatostatin expressing interneuron (**B**). Ten stimuli were delivered at 10 Hz to characterize strength and short-term dynamics of thalamocortical (row 2) and intracortical (rows 3 and 4) synaptic connections (see schematic connectivity diagrams to the left). Note synaptic depression in A2-4 versus facilitation in B2-4. Reproduced with permission from Feldmeyer et al. [5]. A2 and B2 reproduced from Tan et al. [20], copyright (2008) National Academy of Sciences

Another process, which contributes to the dynamics of cortical function, is short-term synaptic plasticity [24]. Upon repetitive activation, excitatory and inhibitory synapses often show facilitation or depression of synaptic efficacy lasting hundreds of milliseconds. Whereas facilitation is attributed to residual elevations in the intracellular calcium concentration during repetitive stimulation, depression is attributed to depletion of synaptic vesicles from the readily releasable pool or to receptor desensitization. Figure 3 shows how the synaptic inputs and outputs of two different types of neocortical GABAergic interneurons differ in their short-term synaptic plasticity. The fast spiking cell shows synaptic depression to repetitive activation of the thalamocortical and intracortical glutamatergic input (Fig. 3A1-3). Furthermore, the GABAergic output of this fast spiking neuron to a PC also reveals synaptic depression (Fig. 3A4). In contrast, the non-fast spiking interneuron shows synaptic facilitation to activation of the thalamocortical and intracortical input (Fig. 3B1-3), and the output of this GABAergic cell to a PC also reveals facilitation (Fig. 3B4). These data indicate that both types of GABAergic interneurons differ in their synaptic input-output function during repetitive network activity, under physiological conditions (e.g., 30–100 Hz gamma oscillations) as well as under pathophysiological conditions (e.g., epileptic seizure). Fast spiking ChCs forming electrical axo-axonic synapses with PCs may even generate high-frequency oscillations (110–160 Hz), which play important roles in higher cognitive function, such as memory formation [21].

## Neocortical dynamics and sensory coding

Neuronal information from the sensory organs activates via parallel pathways the thalamus and then reaches the corresponding primary sensory cortical area. Here the information is processed along vertical and horizontal connections in different micronetworks consisting of inhibitory and excitatory neurons. Currently, we do not completely understand how the sensory information is coded in the neocortical network by single neurons, distinct networks, and specific activity patterns [6]. However, over the last years, we gained important insights into the network properties and their underlying mechanisms. (i) Projections from FO thalamus make soma-near reliable and fast synapses predominantly in L4 and carry specific sensory or motor information. In contrast, inputs from HO thalamus terminate in L1 and L5a on apical dendrites of PCs and increase their excitability. (ii) Excitatory neurons make short- and long-range recurrent connections with other neurons of their own class (mostly not shown in Fig. 1), forming excitatory loops to enhance and prolong neuronal activity. (iii) The genetic, morphological, and electrophysiological diversity of GABAergic interneurons is highly complex and specific interneurons are important for neocortical gain control, plasticity, precise timing of PC discharge, synchronizing and generation of oscillatory activity, and sensory feature selectivity [22]. For example, L4 inhibitory interneurons have been identified as the cells carrying substantial information about specific biophysical properties of the sensory stimulus [15]. (iv) Recently, it has been demonstrated that active behavior (e.g., locomotion) changes the operating mode of the neocortical network and that distinct interneurons play key roles in this modulatory effect [6].

More sophisticated experimental studies in behaving animals are required to address the central question how sensory information is coded in the neocortical network. Extracellular simultaneous recordings from thousands of neurons and fast cellular imaging, both in combination with cell-specific experimental manipulations (e.g., optogenetics), became recently available and already provided surprising insights into the complex interactions in neocortical networks. The canonical microcircuit, as proposed by Douglas and colleagues in 1989 [3], was an important starting point on the way to understand the function of the neocortical circuit. Future studies will show how this network will operate during different functional and behavioral states and how non-neuronal cells (e.g., astrocytes) influence processing in the neocortical network.

## Data Availability

Not applicable.
